# Supplementary material for: Mitochondrial aconitase suppresses immunity by modulating oxaloacetate and the mitochondrial unfolded protein response
Source: Nat Commun. 2023 Jun 22;14:3716. doi: 10.1038/s41467-023-39393-6 (PMC10287738; doi:10.1038/s41467-023-39393-6)
Supplement: Supplementary file 1 — Supplementary Information [file 41467_2023_39393_MOESM1_ESM.pdf]

Supplementary information

Supplementary Figures

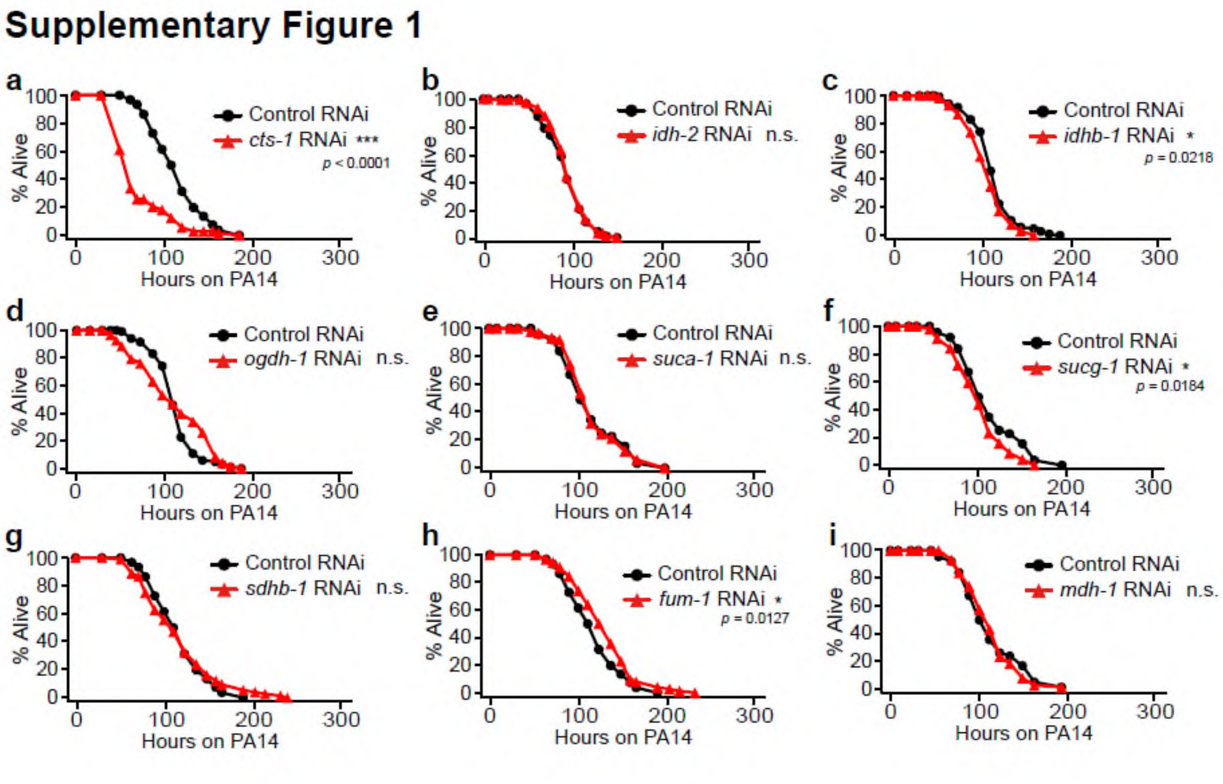

Supplementary Figure 1. The effect of RNAi targeting Krebs cycle enzymes on pathogen resistance. Survival curves from an RNAi screen targeting genes that encode Krebs cycle enzymes, *cts-1* (**a**), *idh-2* (**b**), *idhb-1* (**c**), *ogdh-1* (**d**), *suca-1* (**e**), *sucg-1* (**f**), *sdhb-1* (**g**), *fum-1* (**h**), and *mdh-1* (**i**). All the survival assays were performed on PA14 small-lawn plates at least twice independently (slow-killing assay). Asterisks indicate the significance of differences (*$p < 0.05$, ***$p < 0.001$). n.s.: not significant. The p values for survival data were calculated using a log-rank (Mantel-Cox method) test.

See Supplementary Data 1 for additional repeats and statistical analysis for the survival assay data shown in this figure. Source data are provided as a Source Data file.

Supplementary Figure 2

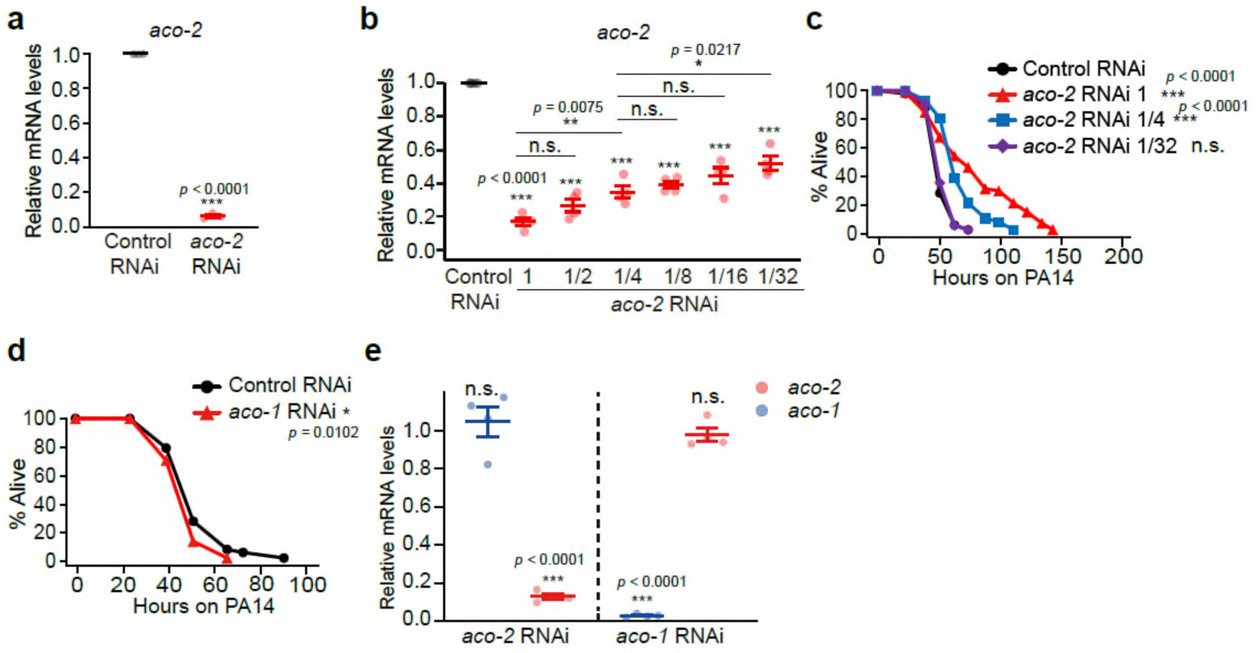

Supplementary Figure 2. Mitochondrial aconitase-mediated immune regulation is distinct from those of cytosolic aconitase.

a, Quantitative RT-PCR data showing that feeding worms with *aco-2* RNAi bacteria reduced *aco-2* mRNA levels (n = 3). **b**, Quantitative RT-PCR data showing the knockdown efficiency of *aco-2* RNAi with serial dilutions (1, 1/2, 1/4, 1/8, 1/16, and 1/32; n = 4). **c**, Differently diluted *aco-2* RNAi (1, 1/4, and 1/32) increased the survival of animals on PA14 in a dose-dependent manner (big-lawn slow-killing assay). **d**, *aco-1* RNAi slightly decreased the survival of animals on PA14 big-lawn plates. In this study, we found that *aco-2* RNAi enhanced the survival of animals on PA14 in both slow- and fast-killing assays. **e**. *aco-2* RNAi reduced the mRNA level of *aco-2* while not altering that of *aco-1*, and *aco-1* RNAi decreased the mRNA level of *aco-1* but did not affect that of *aco-2* (n = 4). For quantitative RT-PCR data, error bars represent the SEM (*$p < 0.05$,

**p < 0.01, ***p < 0.001, n.s.: not significant, two-tailed Student's *t*-test). *ama-1* and *tba-1* mRNA levels were used as normalization controls. See Supplementary Data 5 for the details of primer sequences. All the survival assays were performed at least twice independently. The asterisks in survival curve panels indicate the significance of differences (*p < 0.05). The *p* values for survival data were calculated using a log-rank (Mantel-Cox method) test. See Supplementary Data 1 for additional repeats and statistical analysis for the survival assay data shown in this figure. Source data are provided as a Source Data file.

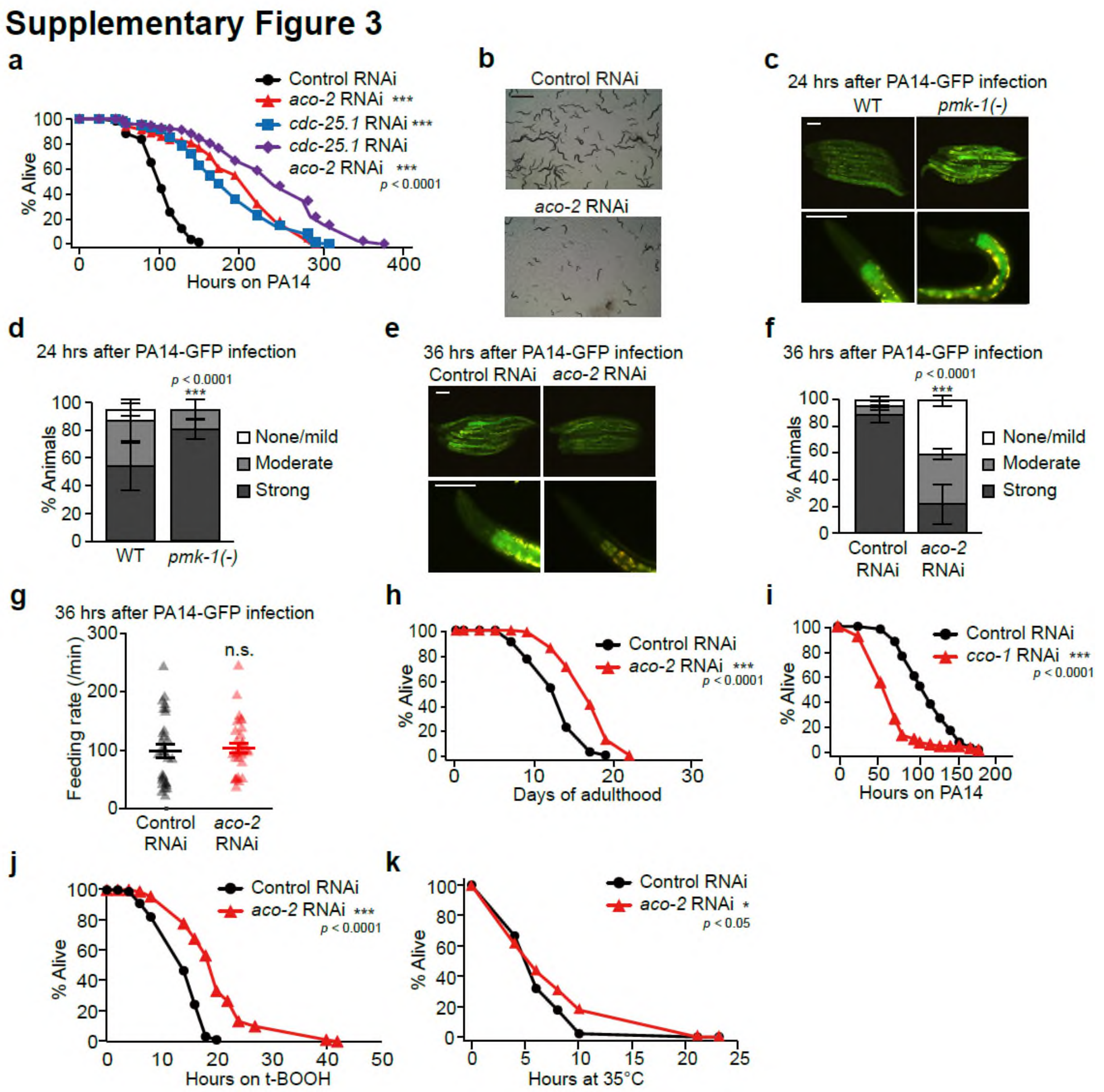

Supplementary Figure 3. Mitochondrial aconitase-mediated immune regulation is different from those regulated by impaired reproduction and by mitochondrial electron transport chain changes.

a, *aco-2* RNAi further increased the survival of *cdc-25.1* RNAi-treated worms that

display sterility, which increases PA14 resistance[1], upon PA14 infection (small-lawn slow-killing assay). **b**, Representative images of control RNAi- and *aco-2* RNAi-treated animals 72 hrs after eggs were placed. Scale bar: 1 mm. *aco-2* RNAi reduced the total brood size of animals and slightly delayed development (three independent trials). **c**,**e**, Representative images of wild-type (WT) and *pmk-1(km25)* [*pmk-1(-)*] animals (**c**) and control RNAi- and *aco-2* RNAi-treated animals (**e**) after PA14-GFP exposure. Scale bar: 100 μm. **d**,**f**, Quantification of PA14-GFP levels in the intestinal lumen in panels **c** and **e**, respectively (N = 30 for WT, N = 27 for *pmk-1(-)* animals, N = 30 for control RNAi- and *aco-2* RNAi-treated animals, three independent trials). Control RNAi-treated animals shown in panels **c** and **d** are the same experimental sets shown in Fig. 1e and 1f. **g**, Feeding (pharyngeal pumping) rates of control RNAi- and *aco-2* RNAi-treated animals 36 hrs after infection with PA14-GFP (N = 30 per condition, three independent trials, n.s.: not significant, two-tailed Student's *t*-test relative to control RNAi). **h**, *aco-2* RNAi extended the lifespan of worms at 25°C, the same temperature for pathogen survival assays, but the effect of *aco-2* RNAi on PA14 resistance is larger than that on lifespan (See Supplementary Data 1 for details). **i**, Knockdown of *cco-1* decreased survival on PA14 (small-lawn slow-killing assay), but substantially increases lifespan[2–4]. These data suggest that *aco-2* RNAi enhances anti-bacterial immunity, distinctly from impaired ETC, which confers longevity but not necessarily increases immunity. **j**,**k**, *aco-2* RNAi increased the resistance of animals against oxidative stress (7.5 mM t-BOOH) (**j**) and against heat stress (35°C) (**k**). All the survival assays were performed at least twice independently. The asterisks indicate the significance of differences (*$p < 0.05$, ***$p <$

0.001, n.s.: not significant), using a log-rank (Mantel-Cox method) test. See Supplementary Data 1 and 2 for additional repeats and statistical analysis for the survival and feeding assay data shown in this figure. Source data are provided as a Source Data file.

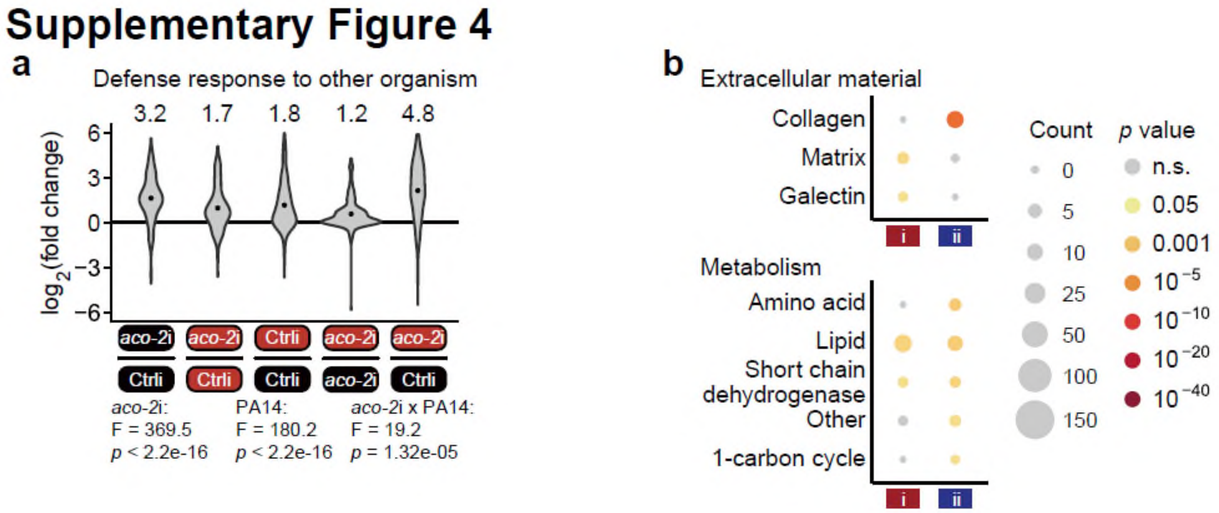

Supplementary Figure 4. *aco-2* RNAi affects the expression of various pathogen response genes.

a, Expression changes conferred by PA14 infection or *aco-2* RNAi in genes associated with GO term “Defense response to other organism (GO:0098542)”. Black dots represent average values. Average fold change is shown on top of each condition. Two-way ANOVA for main effect and interaction. F: between-group variability divided by within-group variability. **b**, Overrepresented WormCat terms, including “Extracellular material” and “Metabolism”, of genes in Group i and ii. *p* values were calculated by using hypergeometric test.

Supplementary Figure 5

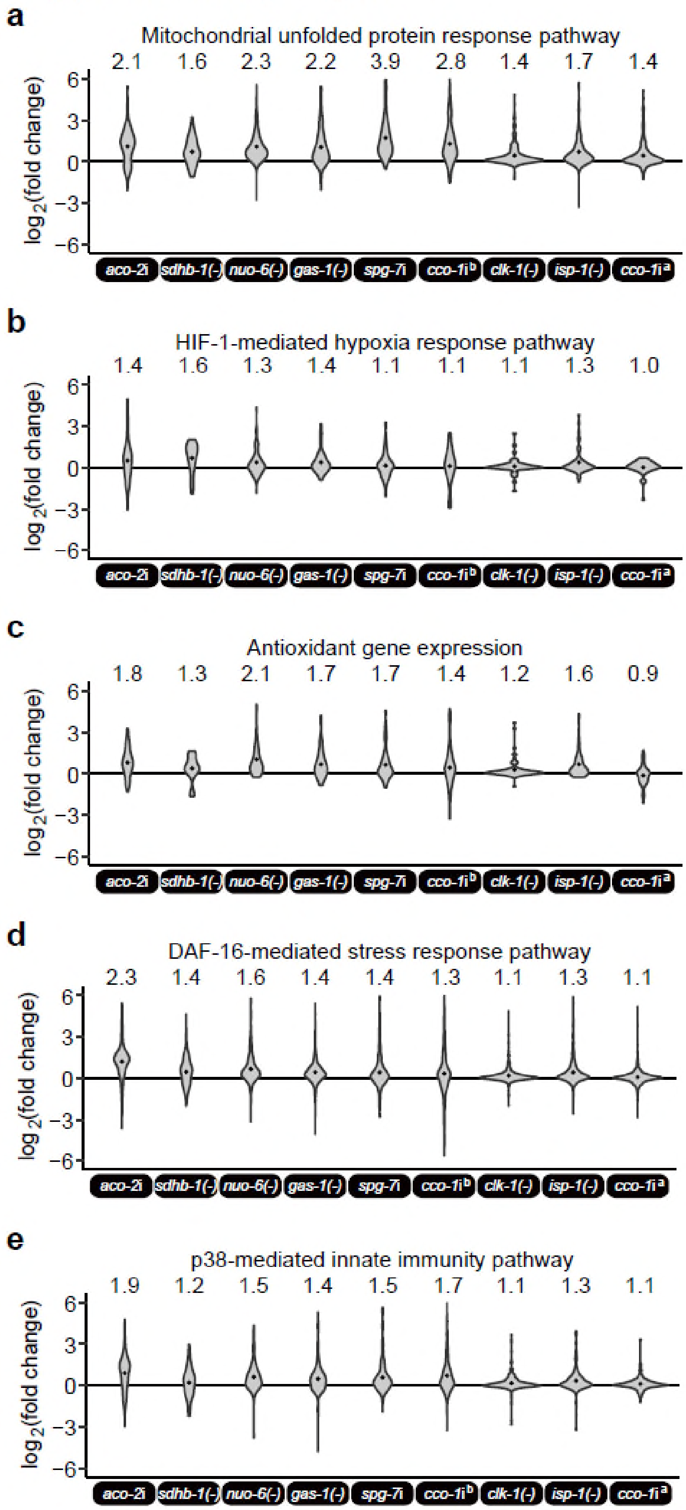

Supplementary Figure 5. Stress response pathways affected by *aco-2* RNAi are different from those by general impairment of mitochondrial functions.

a–**e**, Comparison of expression changes caused by *aco-2* RNAi and those by other mitochondrial dysfunctions for genes associated with multiple stress response pathways[5]: the mitochondrial unfolded protein response pathway (UPR^{mt}) (**a**), the HIF-1-mediated hypoxia response pathway (**b**), the antioxidant gene expression (**c**), the DAF-16-mediated stress response pathway (**d**), and the p38-mediated innate immunity pathway (**e**). Black dots represent average values. Average fold change is shown on top of each condition. We found that upregulation of the three stress response pathways, the UPR^{mt}, the HIF-1-mediated hypoxia response pathway, and the antioxidant gene expression, by *aco-2* RNAi was not the highest among the comparison. For example, our analysis indicated that *spg-7* RNAi conferred the highest upregulation of the UPR^{mt} among the comparisons; this is consistent with our qRT-PCR results of *abf-2* and *hsp-6* (Supplementary Figure 10b,c). In addition, *sdhb-1(R244H)* [*sdhb-1(-)*] conferred the highest upregulation of the HIF-1-mediated hypoxia response pathway among the comparisons; this is consistent with the report showing the pronounced effects of the inhibition of SDH on HIF-1α accumulation in the cytosol[6]. We also found that *nuo-6(qm200)* [*nuo-6(-)*] elicited the highest induction of the antioxidant gene expression. We then showed that upregulation of the DAF-16-mediated stress response pathway and the p38-mediated innate immunity pathway caused by *aco-2* RNAi was the highest among the comparisons that we executed. Overall, these data provide an additional line of evidence for the specific effects of *aco-2* RNAi on pathogen responses. See Legends

of Fig 2, Supplementary Fig 9, and Supplementary Data 3 for specific references of data sets used in these analyses.

Supplementary Figure 6

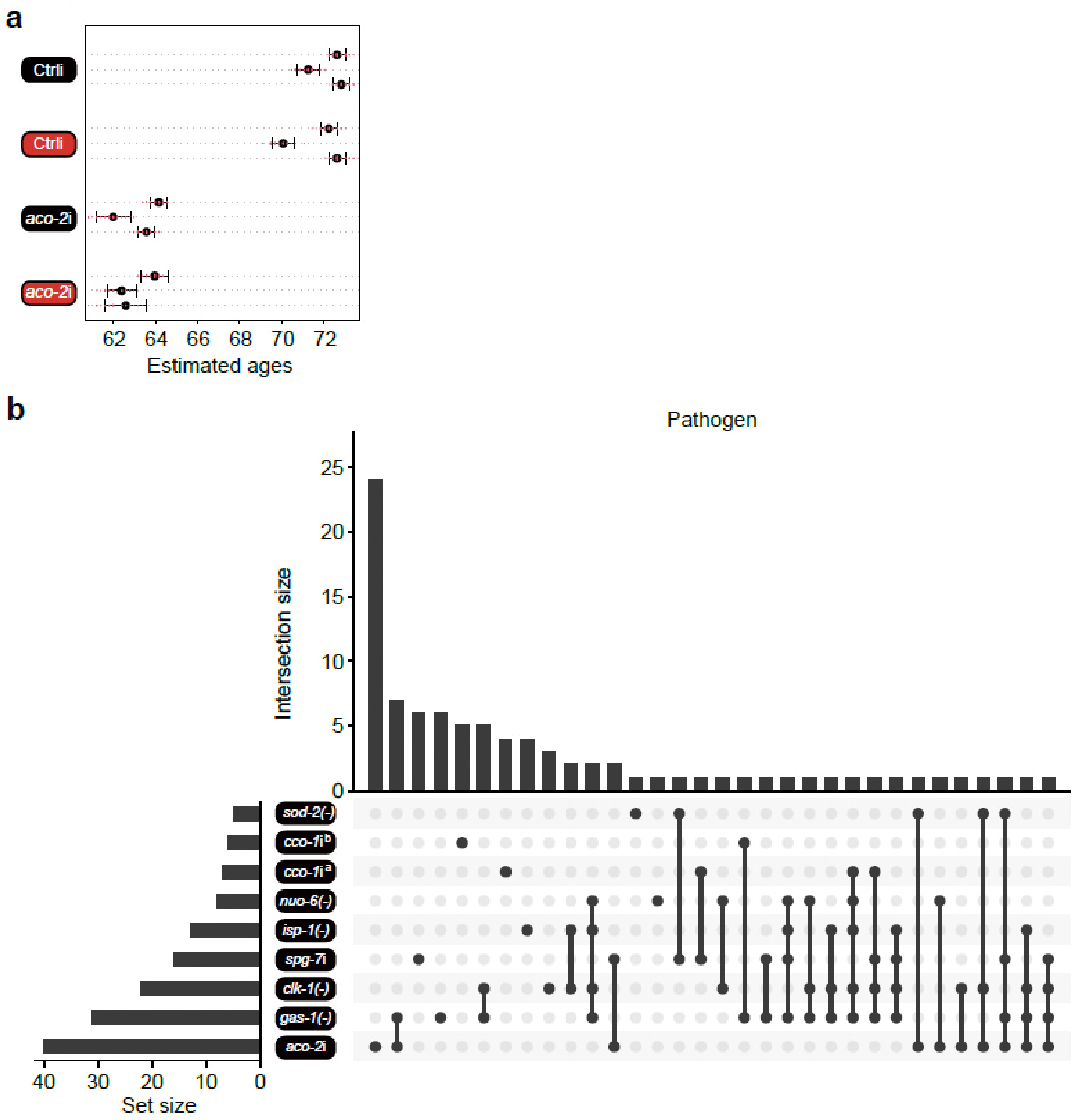

Supplementary Figure 6. *aco-2* RNAi affects pathogen responses that are different from general impairment of mitochondrial functions.

a, Developmental time of samples estimated from RNA seq data by using RAPToR [Real Age Prediction from Transcriptome staging on Reference[7]]. Developmental time of samples was adjusted to that of the control samples (n = 3). A confidence interval is

given by the median absolute deviation (MAD) of bootstrap estimates (estboot) from the global estimate (est), and the resolution of the interpolation (res, time interval between two points of the interpolated reference): [est − (median(|est − estboot|) + res/2); est + (median(|est − estboot|) + res/2)]. **b**, Comparisons between genes upregulated by *aco-2* RNAi and other mitochondrial dysfunctions, among genes associated with WormCat term “Pathogen” of “Stress response”. We found that 24 out of 40 genes (60.0%) that were upregulated by *aco-2* RNAi were not upregulated by other mitochondrial dysfunctions. In contrast, only three genes (*T24B8.5*, *C49G7.5*, and *F22H10.2*) were upregulated by four conditions, which are the maximum common conditions, including *aco-2* RNAi. These data are consistent with our functional data showing the specific role of ACO-2 in the pathogen responses. See Supplementary Data 4 for the details of the gene sets.

Supplementary Figure 7

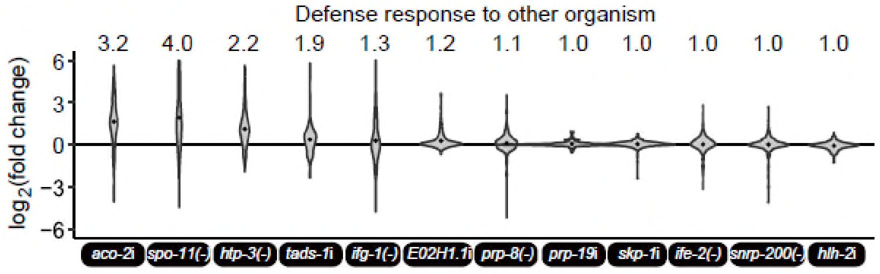

Supplementary Figure 7. *aco-2* RNAi affects pathogen responses distinctly from the general impairment of essential processes.

Comparison of expression changes caused by *aco-2* RNAi and those by genetic inhibition of essential processes for genes associated with GO term “Defense response to other organism (GO:0098542)”: cell proliferation (*spo-11*, *htp-3*, and *tads-1*) (GSE199326; GSE110838), transcription (*hlh-2*) (GSE110835), RNA processing (*E02H1.1*, *prp-8*, *prp-19*, *skp-1*, and *snrp-200*) (GSE175363; Ref[8]; GSE110836), and translation (*ife-2* and *ifg-1*)[9,10]. Black dots represent average values. Average fold change is shown on top of each condition. Disruption of the majority of essential processes, including transcription, RNA processing, and translation, with the exception of cell proliferation, had small effects on the “Defense response to other organism”.

Supplementary Figure 8

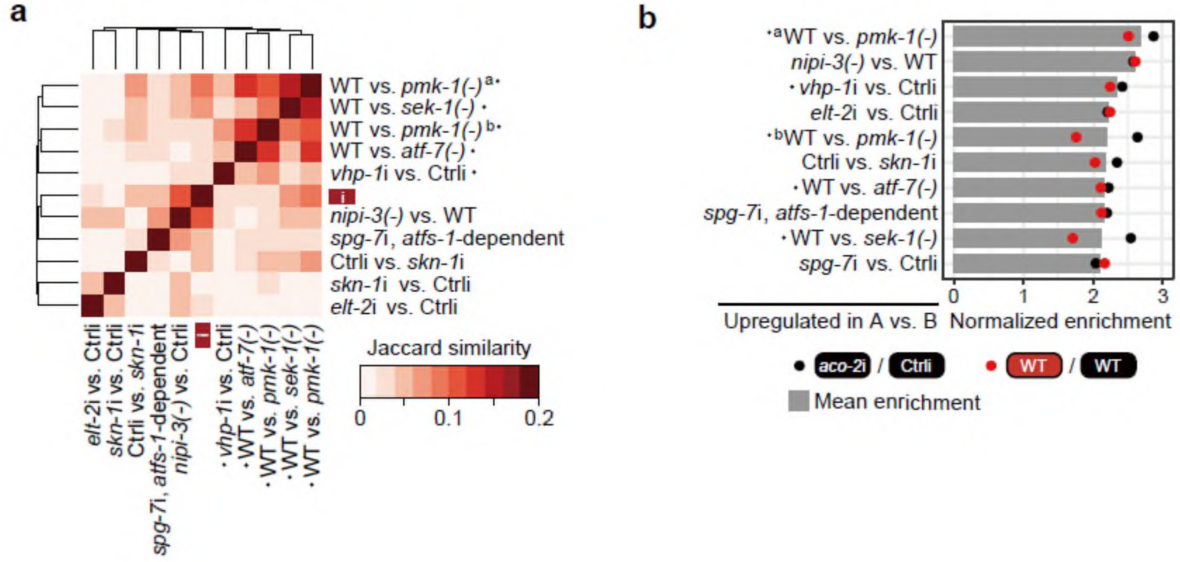

Supplementary Figure 8. *aco-2* RNAi upregulates immune responses via the PMK-1 signaling axis and/or ATFS-1.

a, Hierarchical clustering of the Group i genes and immune signaling gene sets based on Jaccard similarity to display the ratio of intersection to union of gene sets. A cluster with Group i was distinguished from another cluster of PMK-1 signaling axis in the two large clusters. **b**, Normalized enrichment of expression changes of immune signaling gene sets conferred by *aco-2* RNAi (*aco-2*i, black) and PA14 infection (red). Gene sets were aligned by a descending order of mean normalized enrichment scores. *q* values were obtained by calculating the false discovery rate corresponding to each normalized enrichment. *q* values were obtained by calculating the false discovery rate corresponding to each normalized enrichment ($q < 0.05$ for all the comparisons). Data sets with PMK-1 signaling axis were marked with black dots. [a]WT vs. *pmk-1(-)*[11], [b]WT vs. *pmk-1(-)*[12]. See Legends of Fig 2, Supplementary Fig 9, and Supplementary Data 3

for specific references of data sets used in these analyses.

Supplementary Figure 9

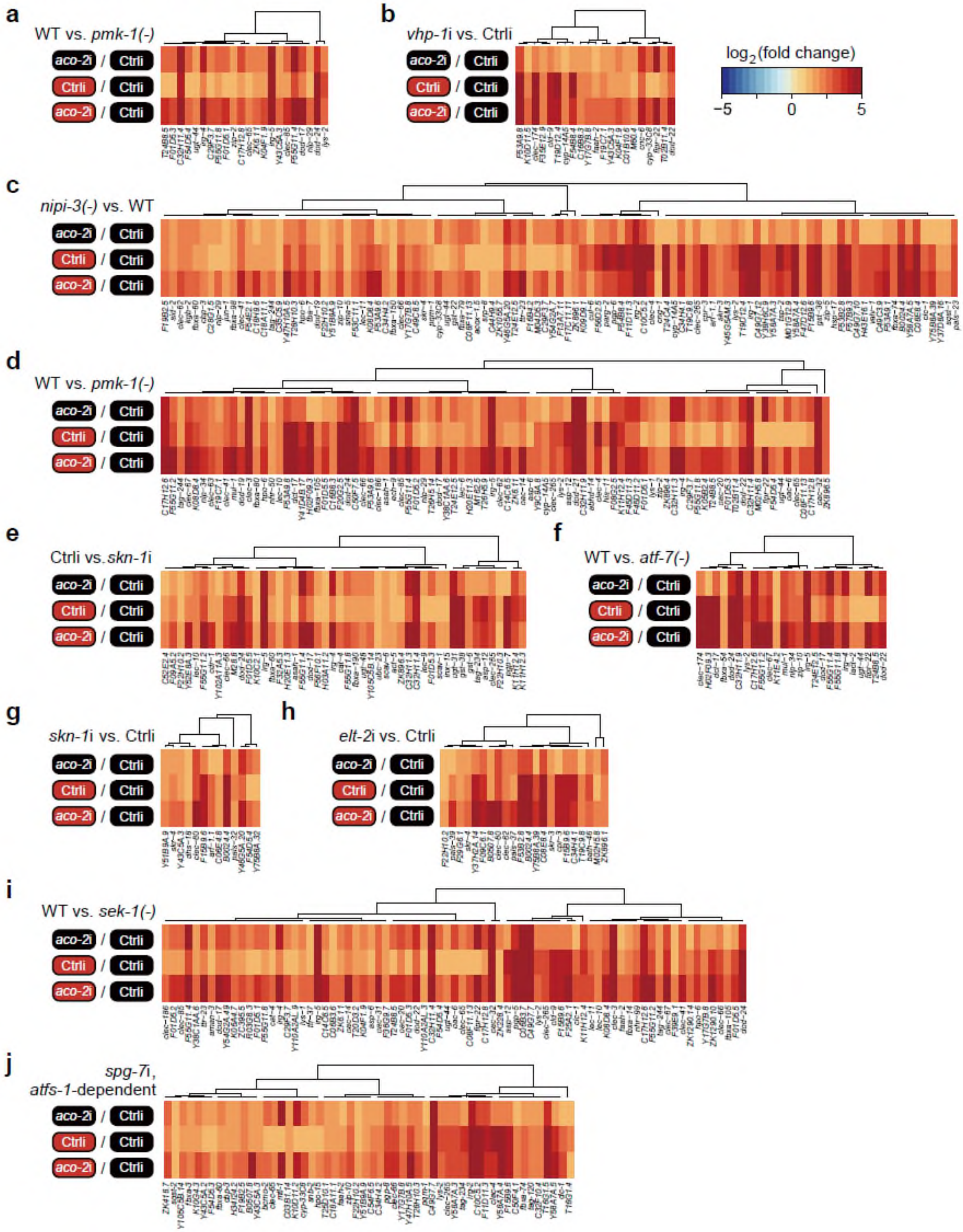

151

Supplementary Figure 9. Knockdown of *aco-2* upregulates genes acting in various immune signaling pathways. Heatmaps showing expression changes in Group i genes caused by PA14 infection or *aco-2* RNAi that overlap with immune signaling genes. Genes upregulated in WT vs. *pmk-1(km25)* [*pmk-1(-)*][12] (**a**), *vhp-1* RNAi (*vhp-1*i) vs. Control RNAi (Ctrli)[a] (**b**), *nipi-3(fr4)* [*nipi-3(-)*] vs. WT[12] (**c**), WT vs. *pmk-1(-)*[11] (**d**), Ctrli vs. *skn-1* RNAi (*skn-1*i)[12] (**e**), WT vs. *atf-7(qd22 qd130)* [*atf-7(-)*][11] (**f**), *skn-1*i vs. Ctrli[13] (**g**), *elt-2* RNAi (*elt-2*i) vs. Ctrli[14] (**h**), and WT vs. *sek-1(km4)* [*sek-1(-)*][15] (**i**). Genes induced by *spg-7* RNAi (*spg-7*i) in an ATFS-1-dependent manner (*atfs-1(tm4525)*)[16] (**j**). Genes were clustered by hierarchical clustering. [a] data (GSE82238) in GEO. See Supplementary Data 3 for the details of the gene sets.

Supplementary Figure 10

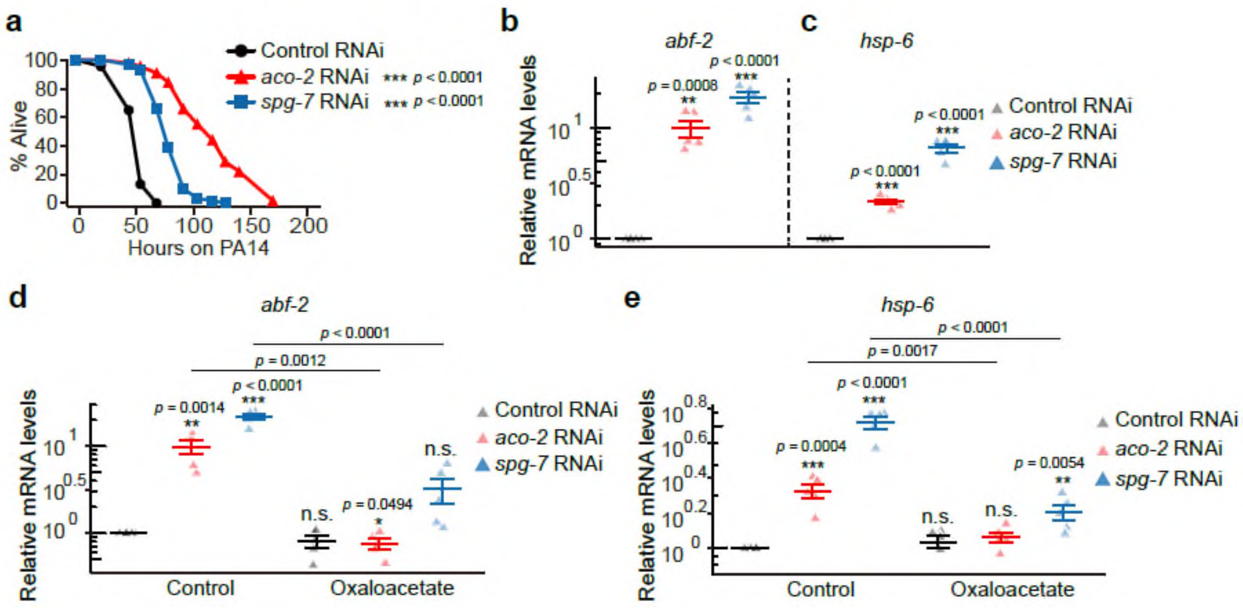

Supplementary Figure 10. Comparison of the effects of *aco-2* RNAi and *spg-7* RNAi on immune responses.

a, The effect of *aco-2* RNAi on the resistance of animals against PA14 was greater than that of *spg-7* RNAi. Asterisks indicate the significance of differences (****p* < 0.001, log-rank (Mantel-Cox method) test relative to control RNAi). **b**,**c**, Upregulation of two selected ATFS-1 targets, *abf-2* (**b**) and *hsp-6* (**c**), by *spg-7* RNAi was stronger than that by *aco-2* RNAi, measured with quantitative RT-PCR (n = 5). Error bars indicate the standard error of the mean (SEM, ***p* < 0.01, ****p* < 0.001, two-tailed Student's *t*-test relative to wild-type animals treated with control RNAi). **d**,**e**, Oxaloacetate robustly decreased the expression of two selected *aco-2* RNAi-induced ATFS-1 targets, *abf-2* (**d**) and *hsp-6* (**e**), upon PA14 infection, while partially suppressing that in *spg-7* RNAi-treated animals, measured by using quantitative RT-PCR (n = 5). Error bars represent

SEM (*p < 0.05, **p < 0.01, ***p < 0.001, n.s.: not significant, two-tailed Student's *t*-test relative to control RNAi under the control condition). *ama-1* and *tba-1* mRNA levels were used as normalization controls. See Supplementary Data 5 for the details of primer sequences. Source data are provided as a Source Data file.

Supplementary Figure 11

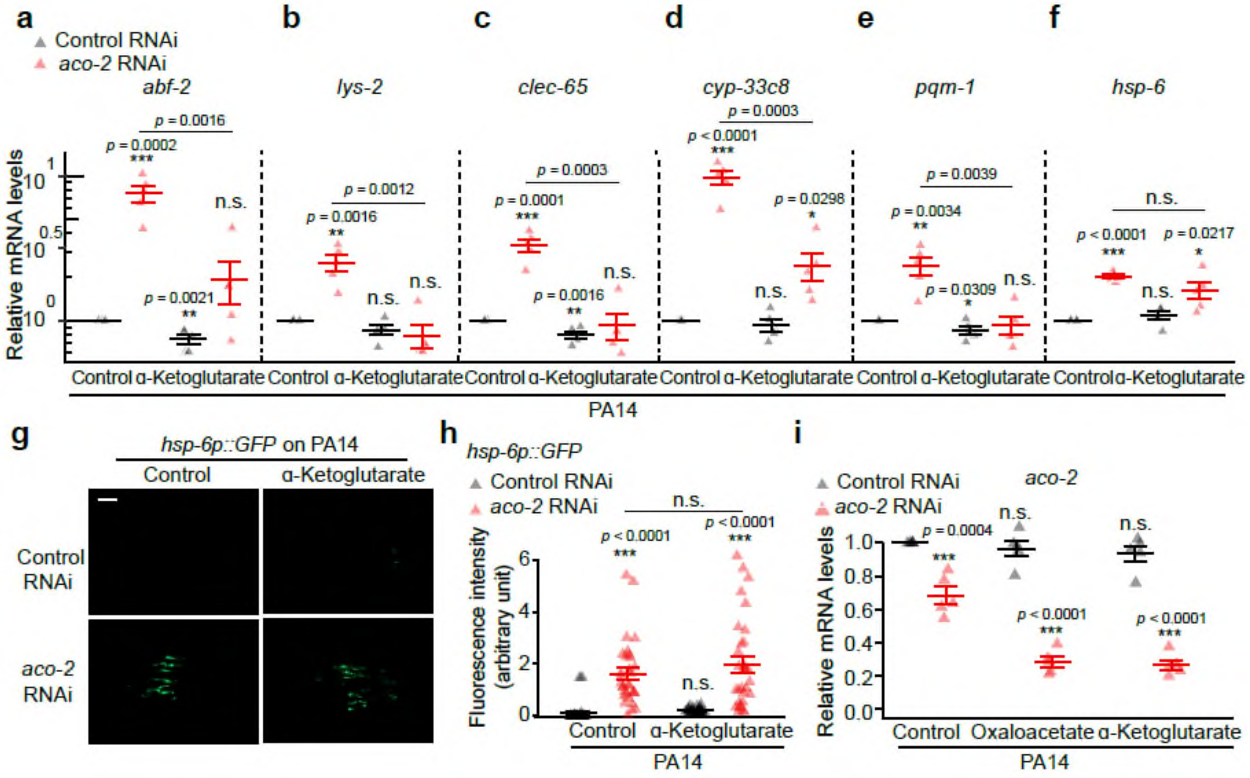

Supplementary Figure 11. α-Ketoglutarate supplementation causes small or no effects on the induction of ATFS-1 target genes by *aco-2* RNAi.

a–**f**, α-Ketoglutarate partially decreased or did not affect the expression of five selected *aco-2* RNAi-induced ATFS-1 targets, *abf-2* (**a**), *lys-2* (**b**), *clec-65* (**c**), *cyp-33c8* (**d**), *pqm-1* (**e**), and *hsp-6* (**f**), upon PA14 infection, measured by using quantitative RT-PCR (n = 5). Error bars represent the standard error of the mean (SEM, **p* < 0.05, ***p* < 0.01, ****p* < 0.001, n.s.: not significant, two-tailed Student's *t*-test relative to control RNAi under the control condition). **g**, Representative fluorescence images of PA14-exposed *hsp-6p::GFP* animals under control RNAi or *aco-2* RNAi conditions with or without (Control)

α-ketoglutarate. Scale bar: 100 μm. **h**, Quantification of the fluorescence intensity of worms in panel **g** (N = 30 for control RNAi in control conditions, and *aco-2* RNAi in α-ketoglutarate-treated conditions, N = 31 for *aco-2* RNAi in control conditions, from three independent trials). Control (control RNAi and *aco-2* RNAi) data shown in panels **g** and **h** are the same experimental sets shown in Fig. 6g,h. **i**, Quantitative RT-PCR data showing that neither oxaloacetate nor α-ketoglutarate supplementation affected the knockdown efficiency of *aco-2* RNAi (n = 5). Error bars indicate the SEM. (***$p < 0.001$, n.s.: not significant, two-tailed Student's *t*-test relative to control RNAi under control conditions). *ama-1* and *tba-1* mRNA levels were used as normalization controls. See Supplementary Data 5 for the details of primer sequences. Source data are provided as a Source Data file.

Supplementary Figure 12

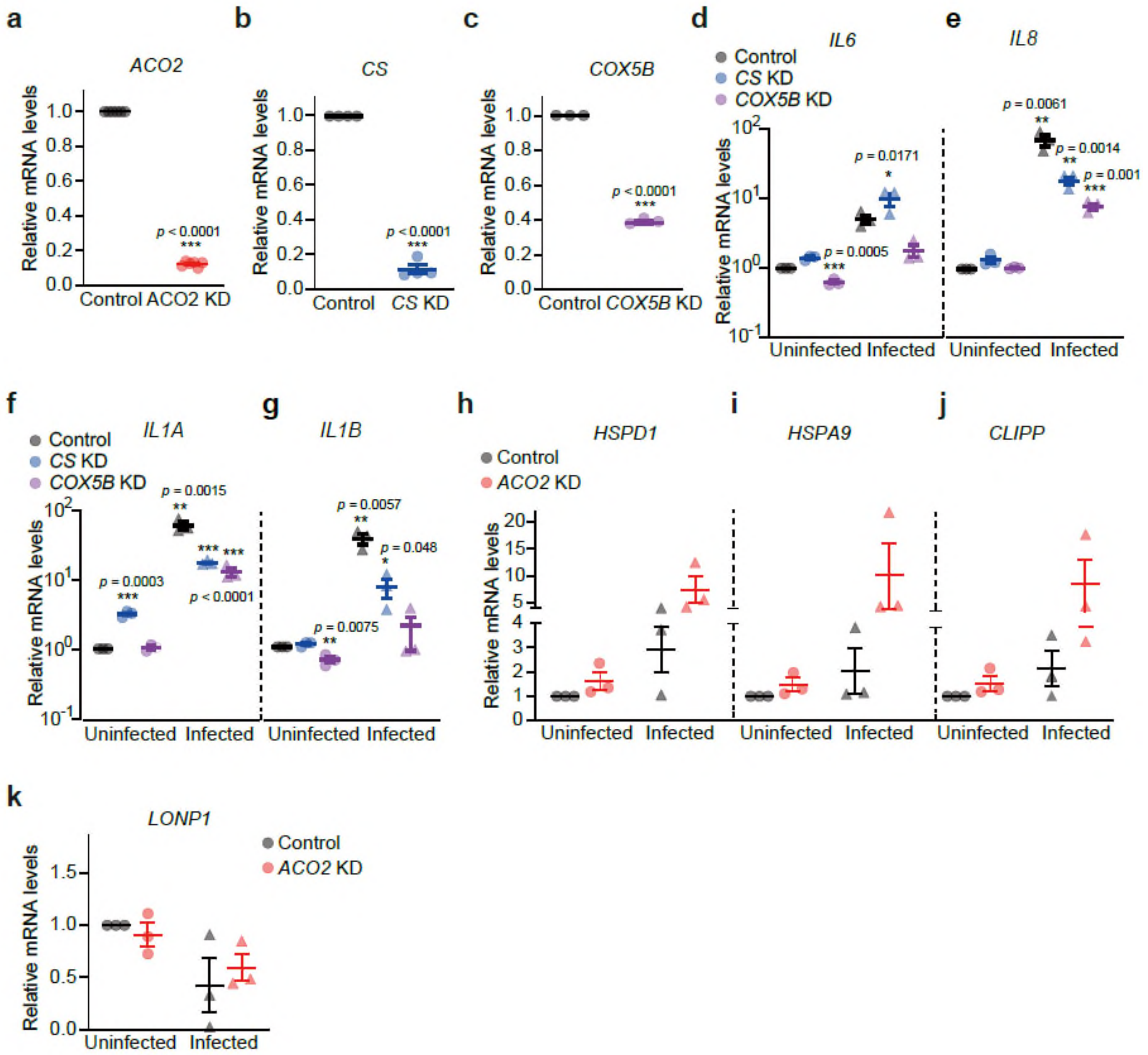

Supplementary Figure 12. siRNA targeting *ACO2*, *CS*, and *COX5B* efficiently downregulated their target mRNA levels, and *ACO2* knockdown increased immunity by upregulating UPR^{mt} in HeLa cells.

a–**c**, siRNA targeting *ACO2* (*ACO2* KD) (**a**), *CS* (*CS* KD) (**b**), and *COX5B* (*COX5B* KD)

(**c**) efficiently decreased *ACO2*, *CS*, and *COX5B* mRNA levels in HeLa cells, respectively (n = 6 for *ACO2* KD, n = 4 for *CS* KD, n = 3 for *COX5B* KD). siRNA against a nontarget (Control) was used as a negative control. **d**–**g**, Relative mRNA levels of *IL6* (**d**), *IL8* (**e**), *IL1A* (**f**), and *IL1B* (**g**) upon *S. aureus* infection in HeLa cells treated with control, *CS* KD, or *COX5B* KD, measured by using quantitative RT-PCR (n = 3). **h**–**k**, Relative mRNA levels of selected ATF5 target genes[17], *HSPD1* (**h**), *HSPA9* (**i**), *CLIPP* (**j**), and *LONP1* (**k**) in HeLa cells treated with control or *ACO2* KD upon *S. aureus* infection were measured by using quantitative RT-PCR (n = 3). Error bars indicate the standard error of the mean (SEM, *$p < 0.05$, **$p < 0.01$, ***$p < 0.001$, two-tailed Student’s *t*-test relative to control conditions). See Supplementary Data 5 for the details of primer sequences. Source data are provided as a Source Data file.

Supplementary Figure 13

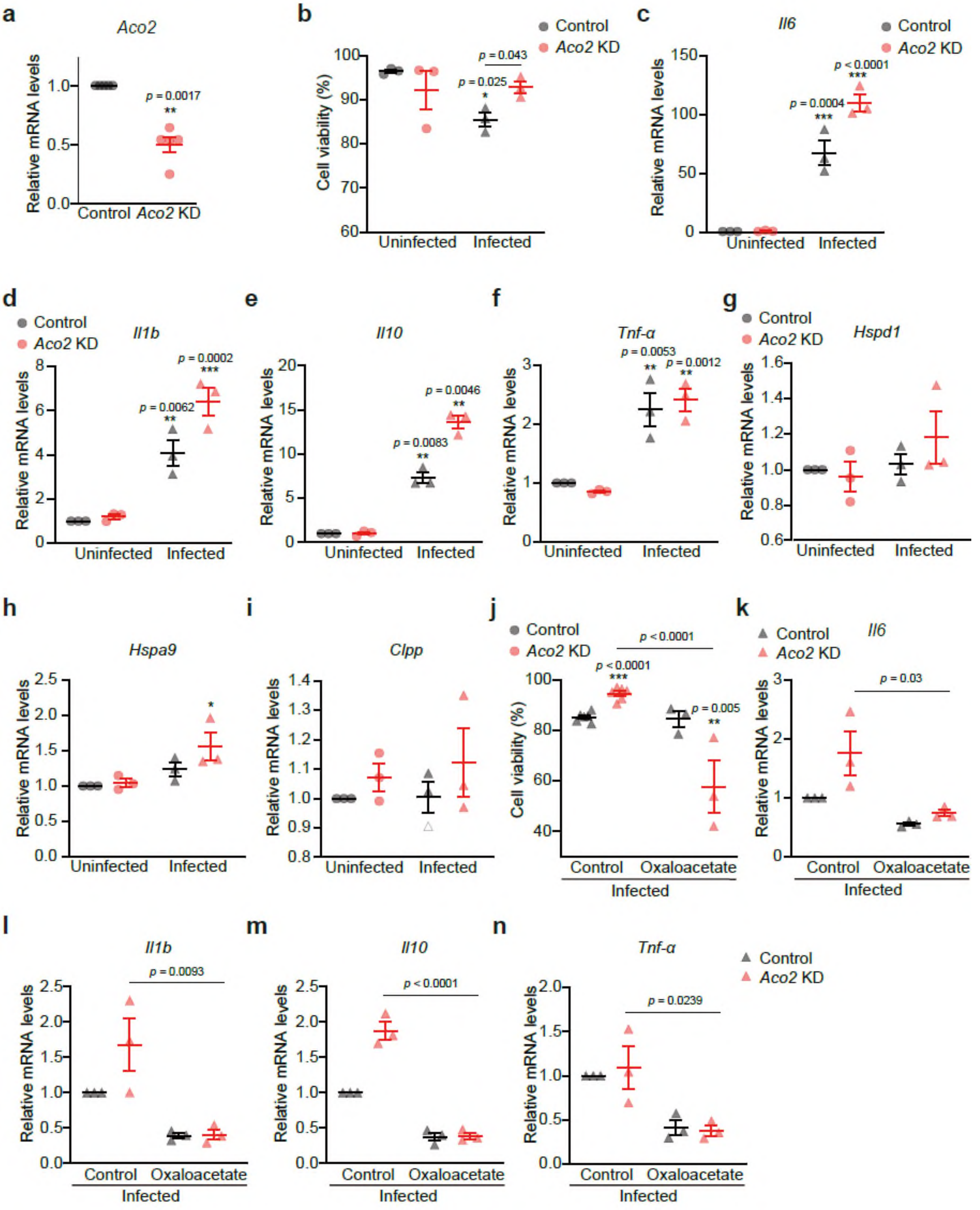

Supplementary Figure 13. Downregulation of *Aco2* increases cellular immunity and UPR^{mt} in macrophage-derived RAW 264.7 cells.

a, siRNA against *Aco2* (*Aco2* KD) efficiently decreased endogenous *Aco2* mRNA levels in RAW 264.7 cells (n = 5). **b**, *Aco2* KD increased the viability of RAW 264.7 cells upon infection with *S. aureus* (n = 3). **c**–**f**, Relative mRNA levels of *Il6* (**c**), *Il1b* (**d**), *Il10* (**e**), and *Tnf-α* (**f**) in RAW 264.7 cells treated with control siRNA (Control) or *Aco2* KD upon infection with *S. aureus*, measured by using quantitative RT-PCR (n = 3). **g**–**i**, Relative mRNA levels of selected ATF5 target genes, *Hspd1* (**g**), *Hspa9* (**h**), and *Clpp* (**i**) were measured by using quantitative RT-PCR in RAW 264.7 cells treated with control or *Aco2* KD upon *S. aureus* infection (n = 3). **j**, Treatment with oxaloacetate suppressed the increased viability of RAW 264.7 cells caused by *Aco2* KD upon infection with *S. aureus* (n = 6 for control, n = 3 for oxaloacetate). **k**–**n**, Relative mRNA levels of *Il6* (**k**), *Il1b* (**l**), *Il10* (**m**), and *Tnf-α* (**n**) upon supplementation with oxaloacetate in *S. aureus*-infected RAW 264.7 cells treated with *Aco2* KD, measured by using quantitative RT-PCR (n = 3). Error bars indicate the standard error of the mean (SEM, *$p < 0.05$, **$p < 0.01$, ***$p < 0.001$, two-tailed Student's *t*-test relative to control conditions). See Supplementary Data 5 for the details of primer sequences.

Supplementary References

1. Tan, M.-W., Mahajan-Miklos, S. & Ausubel, F. M. Killing of *Caenorhabditis elegans* by *Pseudomonas aeruginosa* used to model mammalian bacterial pathogenesis. *Proc. Natl. Acad. Sci.* **96**, 715–720 (1999).
2. Dillin, A. *et al.* Rates of behavior and aging specified by mitochondrial function during development. *Science* **298**, 2398–401 (2002).
3. Lee, S. S. *et al.* A systematic RNAi screen identifies a critical role for mitochondria in *C. elegans* longevity. *Nat. Genet.* **33**, 40–48 (2003).
4. Rea, S. L., Ventura, N. & Johnson, T. E. Relationship Between Mitochondrial Electron Transport Chain Dysfunction, Development, and Life Extension in *Caenorhabditis elegans*. *PLoS Biol.* **5**, e259 (2007).
5. Soo, S.K., Traa, A., Rudich, Z.D., Moldakozhayev, A., Mistry, M., and Van Raamsdonk, J.M. (2022). Genetic basis of enhanced stress resistance in long-lived mutants highlights key role of innate immunity in determining longevity. *Aging Cell*, e13740. 10.1111/acel.13740.
6. Selak, M.A., Armour, S.M., MacKenzie, E.D., Boulahbel, H., Watson, D.G., Mansfield, K.D., Pan, Y., Simon, M.C., Thompson, C.B., and Gottlieb, E. (2005). Succinate links TCA cycle dysfunction to oncogenesis by inhibiting HIF-alpha prolyl hydroxylase. *Cancer Cell* **7**, 77-85. 10.1016/j.ccr.2004.11.022
7. Bulteau, R. & Francesconi, M. Real age prediction from the transcriptome with RAPToR. *Nat. Methods* **19**, 969–975 (2022).
8. Cartwright-Acar, C.H., Osterhoudt, K., Suzuki, J.M.N.G.L., Gomez, D.R., Katzman, S., and Zahler, A.M. (2022). A forward genetic screen in *C. elegans* identifies conserved residues of spliceosomal proteins PRP8 and SNRNP200/BRR2 with a role in maintaining 5′ splice site identity. *Nucleic Acids Res*. **50**, 11834–11857. 10.1093/nar/gkac991.
9. Soo, S.K., Traa, A., Rudich, P.D., Mistry, M., and van Raamsdonk, J.M. (2021). Activation of mitochondrial unfolded protein response protects against multiple exogenous stressors. *Life Sci. Alliance* **4**, 1–16. 10.26508/lsa.202101182.
10. Chomyshen, S.C., Tabarraei, H., and Wu, C.W. (2022). Translational suppression via IFG-1/eIF4G inhibits stress-induced RNA alternative splicing in *Caenorhabditis elegans*. *Genetics* **221**. 10.1093/genetics/iyac075.
11. Fletcher, M., Tillman, E. J., Butty, V. L., Levine, S. S. & Kim, D. H. Global transcriptional regulation of innate immunity by ATF-7 in *C. elegans*. *PLOS Genet.* **15**, e1007830 (2019).
12. McEwan, D. L. *et al.* Tribbles ortholog NIPI-3 and bZIP transcription factor CEBP-1 regulate a *Caenorhabditis elegans* intestinal immune surveillance pathway. *BMC Biol.* **14**, 105 (2016).
13. Oliveira, R. P. *et al.* Condition-adapted stress and longevity gene regulation by *Caenorhabditis elegans* SKN-1/Nrf. *Aging Cell* **8**, 524–541 (2009).
14. Mann, F. G., Van Nostrand, E. L., Friedland, A. E., Liu, X. & Kim, S. K.

Deactivation of the GATA Transcription Factor ELT-2 Is a Major Driver of Normal Aging in *C. elegans*. *PLOS Genet.* **12**, e1005956 (2016).

15. Wu, Z. *et al.* Dietary Restriction Extends Lifespan through Metabolic Regulation of Innate Immunity. *Cell Metab.* **29**, 1192-1205.e8 (2019).
16. Nargund, A. M., Pellegrino, M. W., Fiorese, C. J., Baker, B. M. & Haynes, C. M. Mitochondrial import efficiency of ATFS-1 regulates mitochondrial UPR activation. *Science* **337**, 587–90 (2012).
17. Fiorese, C. J. *et al.* The Transcription Factor ATF5 Mediates a Mammalian Mitochondrial UPR. *Curr. Biol.* **26**, 2037–2043 (2016).
