# Supplementary material for: Mitochondrial aconitase suppresses immunity by modulating oxaloacetate and the mitochondrial unfolded protein response
Source: Nat Commun. 2023 Jun 22;14:3716. doi: 10.1038/s41467-023-39393-6 (PMC10287738; doi:10.1038/s41467-023-39393-6)
Supplement: Supplementary file 3 — Description of Additional Supplementary Files [file 41467_2023_39393_MOESM3_ESM.pdf]

Title: Supplementary Data 1.

Description: Statistical analysis of survival assay data and additional repeats.

Survival assay data within the double-solid lines are obtained from same experimental sets and biological repeats are distinguished by single-solid lines. All *p* values for RNAi experiments were calculated against wild-type animals treated with control RNAi. *aco-2* RNAi extended the lifespan of worms at 25°C, the same temperature for pathogen survival assays (Supplementary Fig. 3h and i); the effect of *aco-2* RNAi on PA14 resistance (46%-102% increase in 12 trials of PA14 small-lawn assays: average 74%) is larger than that on lifespan (20%-31% increase in two trials: average 26%). WT: wild-type, [pmk1]: *p* value against *pmk-1(km25)* treated with control RNAi, [tir1]: *p* value against *tir-1(tm3036)* treated with control RNAi, [aco1]: *p* value against *aco-1(jh131)* treated with control RNAi, [skn1]: *p* value against *skn-1(zj15)* treated with control RNAi, [atfs1]: *p* value against *atfs-1(gk3094)* treated with control RNAi, [zip2]: *p* value against *zip-2(tm4067)* treated with control RNAi, [atf7]: *p* value against *atf-7(qd22 qd130)* treated with control RNAi, [daf16]: *p* value against *daf-16(mu86)* treated with control RNAi, [hsf1]: *p* value against *hsf-1(sy441)* treated with control RNAi, [hlh30]: *p* value against *hlh-30(tm1978)* treated with control RNAi, [aco2i]: *p* value against wild-type treated with *aco-2* RNAi, [cco1i]: *p* value against wild-type treated with *cco-1* RNAi.

Title: Supplementary Data 2.

Description: Statistical analysis of feeding assay data shown in Fig. 1 and Supplementary Fig. 3.

Feeding assay data within the double-solid lines were obtained from same experimental sets. All *p* values for RNAi experiments were calculated against wild-type worms treated with control RNAi. WT: wild-type

Title: Supplementary Data 3.

Description: The list of gene sets related to the immune signaling pathways.

GEO: Gene expression omnibus, GSE: GEO series, PUMA: Princeton University microarray database. DESeq2 and limma indicate that raw RNA-seq and microarray data were reanalyzed to identify differentially expressed genes by using these tools, respectively. Gene sets significantly enriched (*p* value < 10^{-3}) in the GO term "Defense response to other organism" were subsequently chosen by using g:Profiler. It is noted that different mitochondrial dysfunctions change lifespan in different ways; *aco-2*i, *cco-1*i, *clk-1(qm30)*, *isp-1(qm150)*, and *nuo-6(qm200)* extend lifespan, whereas *gas-1(fc21)* and *sdhb-1(R244H)* shorten lifespan. Group i genes substantially overlapped with genes that were downregulated by depletion of *elt-2* and *nipi-3*, but did not with genes that were upregulated by depletion of *elt-2* or *nipi-3*, which are more likely immune signaling genes. We therefore focused on

functionally characterizing other immune regulators in Figures 3 and 4, such as *pmk-1*, *atfs-1*, and *skn-1*, instead of *elt-2* and *nipi-3*. Data sets with PMK-1 signaling axis were marked with black dots.

Title: Supplementary Data 4.
Description: The list of genes upregulated by mitochondrial dysfunctions and related to "Pathogen" of "Stress response" in WormCat.
GEO: Gene expression omnibus, GSE: GEO series. RAPToR indicate that RNA-seq data were reanalyzed to adjust different stages by using the tool. 0: Not significatly changed, 1: Upregulated.

Title: Supplementary Data 5.
Description: List of qRT-PCR primers used in this study.

Title: Supplementary Data 6.
Description: Mass spectrometric parameters of Krebs cycle metabolites.

Title: Supplementary Data 7.
Description: Analysis of Krebs cycle metabolites in Fig. 5a.
The *p* values were calculated by two-tailed Wilcoxon rank sum exact test.
