# Supplementary material for: Mitochondrial aconitase suppresses immunity by modulating oxaloacetate and the mitochondrial unfolded protein response
Source: Nat Commun. 2023 Jun 22;14:3716. doi: 10.1038/s41467-023-39393-6 (PMC10287738; doi:10.1038/s41467-023-39393-6)
Supplement: Supplementary file 11 — Reporting Summary [file 41467_2023_39393_MOESM11_ESM.pdf]

nature portfolio

Corresponding author(s): Seung-Jae V. Lee, Adam Antebi

Last updated by author(s): Apr 27, 2023

Reporting Summary

Nature Portfolio wishes to improve the reproducibility of the work that we publish. This form provides structure for consistency and transparency in reporting. For further information on Nature Portfolio policies, see our Editorial Policies and the Editorial Policy Checklist.

Statistics

For all statistical analyses, confirm that the following items are present in the figure legend, table legend, main text, or Methods section.

n/a	Confirmed	
☐	☒	The exact sample size (*n*) for each experimental group/condition, given as a discrete number and unit of measurement
☐	☒	A statement on whether measurements were taken from distinct samples or whether the same sample was measured repeatedly
☐	☒	The statistical test(s) used AND whether they are one- or two-sided<br>*Only common tests should be described solely by name; describe more complex techniques in the Methods section.*
☐	☒	A description of all covariates tested
☐	☒	A description of any assumptions or corrections, such as tests of normality and adjustment for multiple comparisons
☐	☒	A full description of the statistical parameters including central tendency (e.g. means) or other basic estimates (e.g. regression coefficient) AND variation (e.g. standard deviation) or associated estimates of uncertainty (e.g. confidence intervals)
☐	☒	For null hypothesis testing, the test statistic (e.g. *F*, *t*, *r*) with confidence intervals, effect sizes, degrees of freedom and *P* value noted<br>*Give P values as exact values whenever suitable.*
☒	☐	For Bayesian analysis, information on the choice of priors and Markov chain Monte Carlo settings
☒	☐	For hierarchical and complex designs, identification of the appropriate level for tests and full reporting of outcomes
☐	☒	Estimates of effect sizes (e.g. Cohen's *d*, Pearson's *r*), indicating how they were calculated

Our web collection on statistics for biologists contains articles on many of the points above.

Software and code

Policy information about availability of computer code

Data collection	Quantitative RT-PCR data were collected by using StepOne Software (version 2.3) installed in StepOne Real-Time PCR system.<br>Western blot images were collected using Image Lab software (version 6.1, Bio-Rad, Contra Costa County, CA, USA).
Data analysis	For survival assays, OASIS (http://sbi.postech.ac.kr/oasis), OASIS2 (https://sbi.postech.ac.kr/oasis2), and GraphPad Prism (version 9 for window, GraphPad Software, San Diego, CA, USA) were used for the log-rank (Manel-Cox method) test.<br>For RNA sequencing, alignment, and quantification, C. elegans genome WBcel235 (ce11), Ensembl transcriptome (release 103), STAR (v.2.7.0e), RSEM (v.1.3.1), DESeq2 (v.1.22.2), RAPToR (v1.1), GSEA (v.3.0), g:Profiler (Nov 02 2022), WormCat (v2.0), and R (v.4.0.2, http://www.r-project.org) were used.<br>For gene expression analysis after quantitative RT-PCR, StepOneTM Software (version 2.3) was used.<br>ImageJ software (v.1.53g, Rasband, W.S., ImageJ, U. S. National Institutes of Health, Bethesda, MD, USA, http://rsb.info.nih.gov/ij/) was used for fluorescence image analysis.<br>Image Lab software (version 6.1, Bio-Rad, Contra Costa County, CA, USA) was used for western blot analysis.<br>For semi-quantitative analysis of metabolite using LC-MS, data were analyzed using Xcalibur (v4.1, Thermo Fisher Scientific, MA, USA) and Trace Finder (v4.0, Thermo Fisher Scientific, MA, USA).<br>Figures were assembled with Adobe Illustrator 2020.

For manuscripts utilizing custom algorithms or software that are central to the research but not yet described in published literature, software must be made available to editors and reviewers. We strongly encourage code deposition in a community repository (e.g. GitHub). See the Nature Portfolio guidelines for submitting code & software for further information.

Data

Policy information about availability of data

All manuscripts must include a data availability statement. This statement should provide the following information, where applicable:

- Accession codes, unique identifiers, or web links for publicly available datasets
- A description of any restrictions on data availability
- For clinical datasets or third party data, please ensure that the statement adheres to our policy

All raw and processed sequencing data generated in this study have been submitted to the NCBI Gene Expression Omnibus (GEO; https://www.ncbi.nlm.nih.gov/geo/) under accession number GSE181546.

Human research participants

Policy information about studies involving human research participants and Sex and Gender in Research.

Reporting on sex and gender	n/a
Population characteristics	n/a
Recruitment	n/a
Ethics oversight	n/a

Note that full information on the approval of the study protocol must also be provided in the manuscript.

Field-specific reporting

Please select the one below that is the best fit for your research. If you are not sure, read the appropriate sections before making your selection.

☒ Life sciences ☐ Behavioural & social sciences ☐ Ecological, evolutionary & environmental sciences

For a reference copy of the document with all sections, see nature.com/documents/nr-reporting-summary-flat.pdf

Life sciences study design

All studies must disclose on these points even when the disclosure is negative.

Sample size	All the experiments were done with at least two independent trials. Standard deviations were calculated from the biological replicates. Sample size estimation based on the power was not performed.
Data exclusions	No data were excluded from the study.
Replication	All the experimental data were obtained from at least two independent biological replicates.
Randomization	The samples were randomly allocated into experimental groups.<br>For untargeted metabolomic analysis, we randomized the sample injection in order to minimize the effect of the instrument signal drift. All attempts at replication were successful.
Blinding	All subcellular localization assays were performed double-blindly by at least two independent researchers.<br>Other experiments, including survival assays in this study, were not performed blindly because of apparent phenotypic differences (e.g. different developmental time or movement) among conditions.

Reporting for specific materials, systems and methods

We require information from authors about some types of materials, experimental systems and methods used in many studies. Here, indicate whether each material, system or method listed is relevant to your study. If you are not sure if a list item applies to your research, read the appropriate section before selecting a response.

Materials & experimental systems

n/a	Involved in the study
☐	☒ Antibodies
☐	☒ Eukaryotic cell lines
☒	☐ Palaeontology and archaeology
☐	☒ Animals and other organisms
☒	☐ Clinical data
☒	☐ Dual use research of concern

Methods

n/a	Involved in the study
☒	☐ ChIP-seq
☒	☐ Flow cytometry
☒	☐ MRI-based neuroimaging

Antibodies

Antibodies used

anti-ACO2 anbody (1:1,000, ab129069, Abcam, Cambridge, UK)
anti-ACO1 antibody (1:1,000, ab183721, Abcam, Cambridge, UK)
anti-GAPDH (1:1,000, G9545, Sigma, St. Louis, MO, USA)
anti-COXIV (1:1,000, 4850S, Cell Signaling Technology, Danvers, MA, USA)
anti-rabbit antibodies (1:5,000, #SA8002, ABfrontier, Seoul, South Korea)
anti-mouse antibodies (1:5,000, #SA8001, ABfrontier, Seoul, South Korea).

Validation

All antibodies used in this study were obtained from commercial sources, which were validated for specificity and species reactivity by the manufacturers. The information is readily available on the website of the manufacturers listed below.

Anti-ACO2 (ab129069, Abcam, Cambridge, UK) has been validated for various assays, including Western blotting. The supplier website (https://www.abcam.com/aconitase-2-antibody-epr8282b-ab129069.html) provides 13 references that used this antibody for Western blotting, immunoprecipitation, and immunocytochemistry. We used anti-ACO2 to validate the siRNA-mediated knockdown of ACO2 in HeLa cells by using Western blotting.
Anti-ACO1 (ab183721, Abcam, Cambridge, UK) has been validated for various assays, including Western blotting. The supplier website (https://www.abcam.com/aconitase-1aco1-antibody-epr72262-ab183721.html) provides species reactivity for human sample and data that used this antibody for Western blotting. We used anti-ACO1 to validate the siRNA-mediated knockdown of ACO1 in HeLa cells by using Western blotting.
Anti-GAPDH (G9545, Sigma, St. Louis, MO, USA) has been validated for various assays, including Western blotting, immunoprecipitation, and immunofluorescence. The supplier website (https://www.sigmaaldrich.com/KR/ko/product/sigma/g9545) provides 624 references that used this antibody for Western blotting, immunoprecipitation, immunocytochemistry. We used anti-GAPDH as a loading control, and a cytosolic marker in HeLa cells for Western blotting.
Anti-COXIV (4850S, Abcam, Cambridge, UK) has been validated for various assays, including Western blotting, immunoprecipitation, immunohistochemistry, and immunofluorescence. The supplier website (https://www.cellsignal.com/products/primary-antibodies/cox-iv-3e11-rabbit-mab/4850) provides 464 references that used this antibody for Western blotting, immunoprecipitation, immunohistochemistry, and immunofluorescence. We used anti-COXIV as a mitochondrial maker in HeLa cells for Western blotting.
Anti-rabbit (SA8002, ABfrontier, Seoul, South Korea) has been validated for Western blotting by supplier and other published papers (https://www.nature.com/articles/s12276-020-00523-5, https://www.nature.com/articles/s41598-021-95933-4#Sec8)
Anti-mouse (SA8001, ABfrontier, Seoul, South Korea) has been validated for Western blotting by supplier and other published papers (https://www.nature.com/articles/s41598-021-95933-4, https://www.nature.com/articles/s12276-020-00523-5).

Eukaryotic cell lines

Policy information about cell lines and Sex and Gender in Research

Cell line source(s)

HeLa cells were obtained from American Type Culture Collection (ATCC). The RAW264.7 cells came from ATCC and were gifted to us by Dr. Suk-Jo Kang's lab.

Authentication

The cells were not authenticated.

Mycoplasma contamination

Cell lines used in this study were negative for mycoplasma contamination.

Commonly misidentified lines
(See ICLAC register)

No commonly misidentified lines were used.

Animals and other research organisms

Policy information about studies involving animals; ARRIVE guidelines recommended for reporting animal research, and Sex and Gender in Research

Laboratory animals

Caenorhabditis elegans hermaphrodites at larval and adult stages were used in this study. These include wild-type N2 (Bristol), IJ575 atfs-1(gk3094) V obtained by outcrossing VC3201 four times to Lee-laboratory N2, IJ130 pmk-1(km25) IV obtained by outcrossing KU25 four times to Lee-laboratory N2, IJ824 tir-1(tm3036) III obtained by oucrossing IG685 four times to Lee-laboratory N2, AU78 agIs219[T24B8.5p::GFP::unc-54-3'UTR; ttx-3p::GFP] III, ZD318 atf-7(qd22 qd130) agIs219[T24B8.5p::GFP::unc-54-3'UTR; ttx-3p::GFP] III, IJ1906 hlh-30(tm1978) IV obtained by outcrossing JIN1375 six times to Lee-laboratory N2, IJ1625 skn-1(zj15) IV obtained by outcrossing QV225 four times to Lee-laboratory N2, CF1042 daf-16(mu86) I, IJ134 zip-2(tm4067) III obtained by outcrossing FX4067 four times to Lee-laboratory N2, CF2495 hsf-1(sy441) I, SJ4100 zcIs13[hsp-6::GFP], and IJ975 aco-1(jh131) X obtained by outcrossing

	KJ550 four times to Lee-laboratory N2. L4 (> 90%) and prefertile young adult (< 10%) stage animals were used for PA14 slow-killing assays. L4 stage larval animals were used for PA14 fast-killing assays, S. aureus killing assays, stress resistance assays, and RNA extraction experiments. Young adult animals were used for lifespan assays and metabolite analysis.
Wild animals	No wild animals were used in this study.
Reporting on sex	Caenorhabditis elegans hermaphrodites at larval and adult stages were used in this study.
Field-collected samples	No field-collected samples were used in this study.
Ethics oversight	No ethics oversight was required.

Note that full information on the approval of the study protocol must also be provided in the manuscript.
